# Space Trumps Time When Talking About Objects

**DOI:** 10.1111/cogs.12719

**Published:** 2019-03-21

**Authors:** Debra Griffiths, Andre Bester, Kenny R. Coventry

**Affiliations:** ^1^ School of Psychology University of East Anglia United Kingdom; ^2^ Bester Labs Newcastle Upon Tyne United Kingdom

**Keywords:** Spatial demonstratives, Distance, Time, Virtual reality

## Abstract

The nature of the relationship between the concepts of space and time in the human mind is much debated. Some claim that space is primary and that it structures time (cf. Lakoff & Johnson, 1980) while others (cf. Walsh, 2003) maintain no difference in status between them. Using fully immersive virtual reality (VR), we examined the influence of object distance and time of appearance on choice of demonstratives (*this* and *that*) to refer to objects. Critically, demonstratives can be used spatially (*this/that red triangle*) and temporally (*this/that month*). Experiment 1 showed a pattern of demonstrative usage in VR that is consistent with results found in real‐world studies. Experiments 2, 3, and 4 manipulated both when and where objects appeared, providing scenarios where participants were free to use demonstratives in either a temporal or spatial sense. Although we find evidence for time of presentation affecting object mention, the experiments found that demonstrative choice was affected only by distance. These results support the view that spatial uses of demonstratives are privileged over temporal uses.

## 1 Introduction

In Newton's laws of motion, distance and time are closely related constructs. In human cognition too, it has been argued that distance and time are deeply coupled, but exactly *how* they are connected is the subject of much debate. A common starting point to explore the relationship between them is the expression of distance and time in language. It has long been recognized that the same terms can be used spatially and temporally. For example, the demonstratives *this* and *that* can refer to objects in space (*this red triangle*,* that green square*) and to objects and events at different points in time (*this month*,* that week*; Diessel, 1999: *this* is in current temporal focus). Similarly prepositions such as *in* and *on* can be used temporally (*I'll see you in an hour*) as well as spatially (*The coffee is in the cup*).

Examination of patterns of language data have been used as evidence for an asymmetrical relationship between space and time. In so‐called cognitive linguistics and embodied theories of language and cognition, space is assumed to be primary, with other senses of linguistic terms emerging from spatial senses/constructs (Lakoff & Johnson, 1980, 1999—“conceptual metaphor theory”; Meier & Robinson, 2004; Schubert, 2005). The developmental trajectories of language learning seem to support such an asymmetric relationship, with spatial senses of terms acquired earlier in development than more “extended” uses of those same terms; this is true both of prepositions (e.g., Clark, 1973) and demonstratives (Küntay & Özyürek, 2006). And it appears to be the case that time is more often talked about in terms of space than the reverse (Lakoff & Johnson, 1980, 1999). However, one must strike a note of caution; the focus on linguistic stimuli is problematic from a conceptual metaphor perspective as the relationships between space and time domains, for example, may simply represent shared descriptive associations rather than deeper conceptual content (Murphy, 1996).

An alternative account of the mapping between space and time comes from non‐linguistic work. Walsh (2003; Bueti & Walsh, 2009), in a synthesis of neurological evidence and magnitude estimation data, offers A Theory of Magnitude (ATOM) in which a common analog magnitude estimation system is proposed to underlie space, time, and number, situated in the parietal cortex. Such a model can account for a range of magnitude estimation parameters elegantly, and it is supported by a wide range of data (e.g., Cai & Connell, 2015; see Lourenco & Longo, 2011, for a useful review). There is, however, some (non‐linguistic) psychophysical data to suggest support for the alternative conceptual metaphor approach, which assumes an asymmetrical relationship between space and time. Casasanto and colleagues (Casasanto & Boroditsky, 2008; Casasanto, Fotakopoulou, & Boroditsky, 2010) got both adults and children to judge lines/dots/snails moving across a screen at varying speeds/durations. They found a greater influence of spatial extent on temporal judgments than temporal extent on spatial judgments. We note that these asymmetries might be at least partly accounted for due to the specifics of the tasks used, and we concur with Lourenco and Longo (2011) that conceptual metaphor theory might struggle to account for some of the considerable body of data supporting ATOM—most notably the fact that one “abstract” domain, number, affects judgments about another abstract domain, duration (Dormal, Seron, & Pesenti, 2006; Droit‐Volet, Clément, & Fayol, 2003), not predicted by conceptual metaphor theory.

Here, we adopt a different approach to the relationship between space and time, returning to language data that originated the conceptual metaphor account. Our focus is on demonstratives (e.g., *this* and *that*)—terms that are among the most significant in all languages. Philologically, demonstratives emerge as the earliest traceable words in languages, they occur in all languages (Deutscher, 2005; Diessel, 1999, 2006), and are among the highest frequency terms in a language. They are also among the first words all children acquire (Clark, 1978, 2003) and are intimately linked with deictic gestures (Clark, 1996; Diessel, 2006). Yet they have been neglected from an empirical point of view, with a notable absence of any empirical work on temporal demonstrative use. Here we pit *where* objects appear (spatially) in a visual array with *when* those objects appear (temporally). This allowed us to test if demonstratives—terms that can critically be used spatially or temporally—are prioritized to make spatial distinctions, temporal distinctions, or a combination of spatial and temporal distinctions. In order to do so, we presented objects in virtual reality (VR) arrays to afford experimental control that is simply not possible using “real” objects. This introduced a secondary goal—to establish if using (spatial) language in virtual space mirrors language use in real space.

### 1.1 VR and spatial language

Virtual environments (VEs) are now commonplace in the entertainment industry and are also being used increasingly as platforms for experiments and simulations of real environments. There has been considerable research into how one perceives space and distance in VE. Results have consistently shown that distances in VEs are underestimated, regardless of measurement methods (see Renner, Velichkovsky, & Helmert, 2013 for a review). The majority of studies have used direct methods such as verbal estimates, perceptual matching, and blind walking, and in most cases examined distances beyond peripersonal (reachable) space. Two notable studies (Geuss, Stefanucci, Creem‐Regehr, & Thompson, 2010; Stefanucci, Creem‐Regehr, Thompson, Lessard, & Geuss, 2015) have examined the accuracy of affordance judgments, such as graspability and whether or not the participant could pass or reach through apertures, in real environments versus VEs. Their results were consistent with an underestimation of distances in VEs, suggesting that misjudgments can affect other aspects of perception. However, few studies have investigated the connection between distance perception and other aspects of perception or language use.

The distinction between peripersonal and extrapersonal space (beyond reach) is an important one that appears to map onto language use in the real world. There is strong evidence that two separate brain systems represent these areas of space (Berti & Rizzolatti, 2002; Cowey, Small, & Ellis, 1994; Halligan & Marshall, 1991; Làdavas, 2002) and that our perceptions, actions, and use of language is affected in the real world by these distinctions (Coventry, Valdés, Castillo, & Guijarro‐Fuentes, 2008; Griffiths & Tipper, 2009; Longo & Lourenco, 2006). Furthermore, it has been demonstrated that, while peripersonal space is defined as reachable space, our perception of peripersonal space is not fixed and can be extended by tool use (Farnè & Làdavas, 2000; Longo & Lourenco, 2006) or contracted (Lourenco & Longo, 2009).

Results from Coventry, Griffiths, and Hamilton (2014) and Coventry et al. (2008) testing the demonstratives *this* and *that* show that there is a strong mapping between spatial perception and the choice of demonstrative, both in the distinction of peripersonal and extrapersonal space, as well as changes with tool use. Their studies show that the use of *this* to refer to objects is dominant in peripersonal space and drops off in a graded manner in extrapersonal space, consistent with results of perceptual experiments mentioned above. The use of *this* is also extended further into extrapersonal space with tool use, suggesting that the use of demonstratives is built upon spatial perception.

With the established mapping between demonstrative use and spatial perception in the real world, it makes the use of demonstratives an excellent paradigm to explore spatial perception and language in VEs. The majority of studies investigating distance perception in VR have examined only extrapersonal space (Creem‐Regehr, Stefanucci, & Thompson, 2015; Renner et al., 2013); the study we report below is one of only a few to look specifically at peripersonal and extrapersonal space in VR. One paper that did compare distance perception in peripersonal and extrapersonal space (Armbrüster, Wolter, Kuhlen, Spijkers, & Fimm, 2008) found underestimations in extrapersonal space but unexpectedly overestimations in peripersonal space. They consider that the use of a projection display placed at the border of peripersonal and extrapersonal space may have produced different parallaxes, which could have influenced estimates. The projector might also have acted as an anchoring point, with estimates being drawn toward the screen. Their study highlights the potential effect the apparatus may have on results.

One key goal in this series of studies is to explore whether the close link between demonstrative use and spatial perception holds in VR and specifically if the use of language in the form of demonstratives in VR matches the use of these terms in the real world.

### 1.2 Overview of method

We adopted the “memory game” method pioneered to elicit naturalistic demonstrative use by Coventry and colleagues (Coventry et al., 2008, 2014; Gudde, Coventry, & Engelhardt, 2016; Gudde, Griffiths, & Coventry, 2018). In this method, an object is positioned on a table at various distances in front of a participant. Believing the experiment to be about the influence of language on memory for object location, participants are instructed to point at the object and name it using a set phrase structure (ostensibly to keep language use consistent across participants). They did so using a three‐word phrase consisting of a demonstrative, the object color, and the object name (*this red square*;* that blue diamond*). This method has previously revealed that spatial demonstrative use in English is affected by the distance an object is from a speaker, the ease of interaction with the object (Coventry et al., 2008), and also a range of object properties including visibility, familiarity, and ownership (Coventry et al., 2014).

## 2 Experiment 1

The goals of this first experiment were two‐fold. First, we wanted to establish if demonstrative use in virtual space directly mirrors demonstrative use in real space. To test this, the environment modeled in VR was the same as the real environment used in earlier studies (Coventry et al., 2008, 2014), thus affording direct comparison. Second, we compared demonstrative use when describing the location of an object in a single versus double object array. It was important to understand if the addition of a second object introduced contrastive demonstrative use—which may be rather different to single object use (see Coventry et al., 2014, for discussion).

### 2.1 Method

#### 2.1.1 Participants

Thirteen participants (four male) were recruited from the student population. Their average age was 24.2 years (range 19–41 years), and all were right‐handed, native mono‐lingual speakers of English. Participants took part for (nominal) payment. All participants had normal or corrected‐to‐normal vision. Stereoacuity was measured using the Randot Stereotest (Stereo Optical Inc, Chicago, IL), and all participants had a threshold of at least 40 arc seconds.

#### 2.1.2 Procedure

Participants were led blindfolded into the room to avoid being cued by the actual dimensions of the real table and room space. For health and safety reasons, an actual table was placed in the room, in case participants should find the virtual image of a table so compelling that they leaned on the table. Participants sat while they were fitted with a NVIS SX60 head‐mounted display unit (through which the virtual images were shown) and the 5DT data glove. The headset was fitted with retroreflective markers to track their head position and movements, using the Naturalpoint Optitrack system. This information was relayed to the Vizard program so that the view participants saw was updated, according to their movements, in real time. The glove was also fitted with markers, and an arm band with markers was placed around the participant's elbow. The markers allowed participants to see a rendering of their hand and arm in the VR environment, including articulated movement of their fingers.

**Figure 1:**
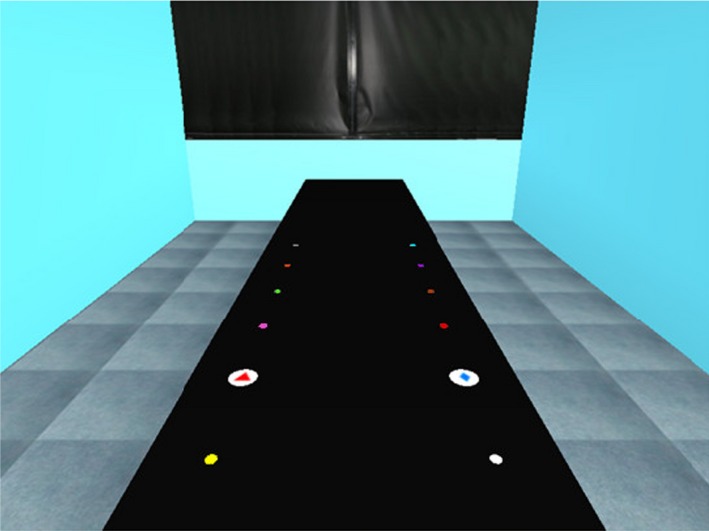
The virtual table used in the experiments.

The VE comprised a table (on which the experimental objects appeared set in a virtual room) (see Fig. 1). Participants were first given a 5 min introduction to accustom them to the VE. They were asked to walk around the table and point to and identify various objects that were shown on and around the table. Participants were then seated at the table.

The virtual table appeared 75 cm wide and 320 cm long. On the table were 12 locations marked by uniquely colored dots, one row of six on the left and another of six on the right (see Fig. 1). The dots in each row were placed at 25 cm intervals away from the participant, starting at 25 cm. The first three locations (25, 50, and 75 cm) in each row were within peripersonal space for all participants (confirmed at the end of the experiment by measurement of reaching distance); the other locations (100, 125, and 150 cm) were beyond participants’ reach. The table appeared in a room with walls and a ceiling. Participants were introduced to the color of each dot.

Participants were informed that they would be taking part in a “memory game” (see Coventry et al., 2008) and that they would be asked questions afterward about the objects that had appeared. In each trial either one or two disks appeared (the disks were colored shapes on a white background), each covering a separate dot location. When two objects appeared, they appeared at the same time. They were told beforehand which object was to be the target (e.g., “The target is the red square” on the first trial, thereafter “Color shape”). They were asked to point at the target object and identify it using a set phrase consisting of a demonstrative, a color, and a shape (e.g., *this red square, that blue diamond*). Once the participant had identified the target, the object(s) were removed from view. Participants were given six practice trials, which ensured that participants always produced the set phrase (including a demonstrative) during the experimental trials.

The manipulations were the distance the target object appeared at (six distances), the side it appeared on (left or right), and whether or not the target appeared with a secondary object. This was a 2 (side) × 2 (distance: peripersonal or extrapersonal) × 2 (number of objects appearing) design. There were 48 trials.

### 2.2 Results and discussion

The percentage use of *this* was calculated for each participant, with the distance collapsed into two regions, peripersonal and extrapersonal space (following Coventry et al., 2008) (see Table 1).

**Table 1:** Percentage of “THIS” responses in each condition across peripersonal and extrapersonal space, split by side

Condition	Target Position	Peripersonal (25–75 cm)		Extrapersonal (100–150 cm)	
		*M*	*SEM*	*M*	*SEM*
Experiment 1					
Single object	Left	53.85	8.26	20.51	6.01
	Right	57.69	8.57	11.54	3.95
Two objects	Left	58.97	8.97	17.95	4.80
	Right	66.67	8.01	17.95	6.39
Experiment 2					
Target first	Left	60.42	8.03	21.88	6.58
	Right	57.29	6.63	22.92	7.28
Target same	Left	60.42	8.03	19.79	5.94
	Right	58.33	8.19	17.71	3.87
Target second	Left	55.21	8.01	21.88	5.21
	Right	54.17	7.22	16.67	4.56
Experiment 3					
Target first	Left	60.00	7.24	25.56	7.43
	Right	70.00	6.13	27.78	8.40
Target same	Left	64.44	4.56	28.89	9.27
	Right	65.56	5.51	20.00	7.49
Target second	Left	67.78	5.26	25.56	6.88
	Right	66.67	6.90	21.11	7.36

**Figure 2:**
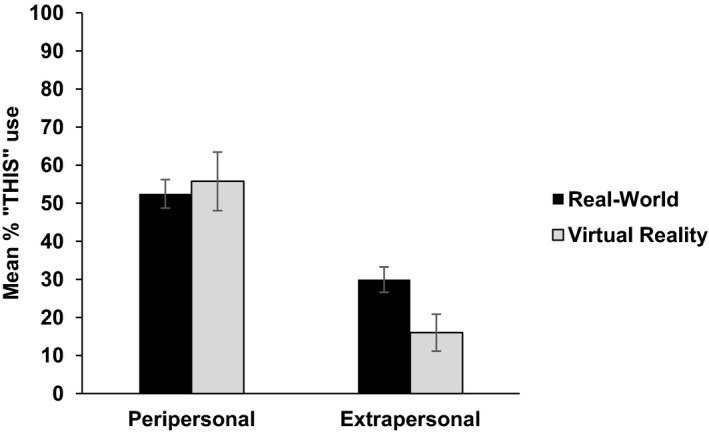
Use of “this” by distance and environment in Experiment 1. Error bars represent standard errors of the mean.

First, we wished to establish whether or not the results in VR matched those found in our previous real‐world studies. We compared the results (percentage *this* usage) of the single object condition from this experiment with the combined results from Coventry et al.'s (2014) language experiments (Experiments 1, 3, and 5). Comparing the results in a 2 (distance: peripersonal or extrapersonal) × 2 (Environment: real vs. VR) anova, we found no main effect of environment, *F*(1, 75) = 0.586, *p* = .446 partial η^2^ = 0.008. There was a significant effect of distance, *F*(1, 75) = 43.926, *p *< .001, partial η^2^ = 0.369. There was also a near‐significant interaction between distance and environment, *F*(1, 75) = 3.358, *p* = .071 partial η^2^ = 0.043, see Fig. 2.

These results indicate that talking about object location in virtual space shows the same pattern of results as talking about object location in real space, where *this* is used significantly more in peripersonal than in extrapersonal space. It supports and extends the results of Coventry et al. (2008) that show that spatial demonstrative use maps onto the distinction between near and far perceptual space,^1^ and that perceptual distinction is evident and influential in virtual space, even though participants do not physically interact with the objects in this virtual world. Such a consistent pattern of results gives us confidence that VR is a suitable platform which to tap language use in controlled settings.

**Figure 3:**
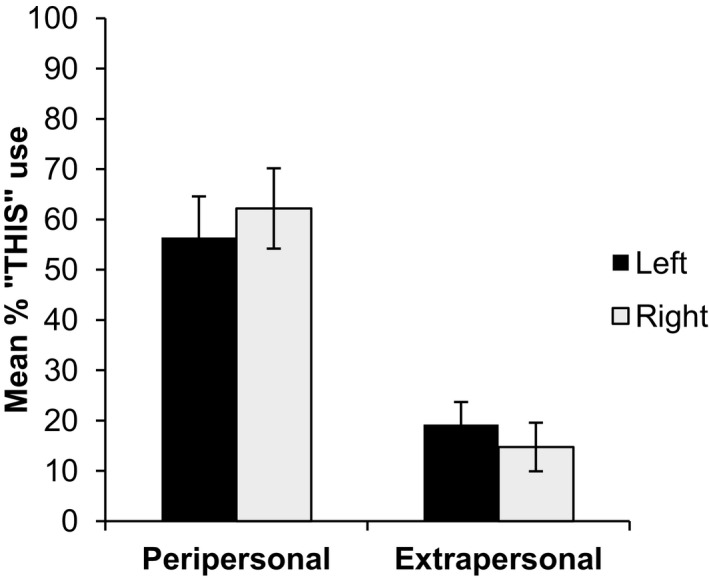
Interaction between distance and side in Experiment 1. Error bars represent standard errors of the mean.

Next, the results from all conditions in VR (including where two objects appeared) were analyzed in a 2 (side) × 2 (distance: peripersonal or extrapersonal) × 2 (number of objects appearing) anova. Overall, there was a clear main effect of distance, *F*(1, 12) = 37.78, *p *< .001, partial η^2^ = 0.759, with participants using *this* more in peripersonal (*M* = 59.29%) than extrapersonal space (*M* = 16.99%). There was no main effect of the side on which the target object appeared, *F*(1, 12) = 0.07, *p* = .790 partial η^2^ = 0.006, but there was, however, an interaction between distance and side, *F*(1, 12) = 5.10, *p* = .043, partial η^2^ = 0.298, Fig. 3. In peripersonal space the use of *this* was higher when the target object appeared on the right side (*M* = 62.18%) than when it appeared on the left (*M* = 56.41%), but not reliably so (*p* = .321, Tukey test). In extrapersonal space the situation was reversed, with the use of *this* being slightly higher when the target appeared on the left (*M* = 19.23%) than the right (*M* = 14.74%), but again this contrast was not significant (*p* = .524). There were no other significant main effects or interactions (*F *< 1.18).

In addition to strong evidence on the importance of the peripersonal/extrapersonal for talking about object location in virtual space, the results also provide tentative evidence of both attentional and action‐based spatial preferences for demonstrative choice. In peripersonal space, where the preferred hand could potentially more easily make contact with the object on the right‐hand side (all the participants were righties), there is a slight preference to use *this* more for the object on the right compared to objects on the left. When the object was placed in extrapersonal space where the objects could not be directly reached, this pattern was reversed, with a tendency to use *this* more for objects on the left compared to objects on the right. We take this to reflect a general left to right processing bias, with *this* associated with the object in a location where attention is first directed.

The results of the first experiment also show that the presentation of a second object with the object that is being talked about does not influence how one uses demonstratives for that object location. This is important, as it allows teasing apart of two possible origins of contrastive use of demonstratives. One account is that mere presence of a second object is the driver for contrastive use. The other account is that contrastive use emerges only when a speaker intends to contrast two objects using language, which is the case when both objects are marked linguistically (*this object and that object*). Our data point to the second account of contrast—we found no differences in demonstrative use as a function of the presence of one or two objects.

## 3 Experiment 2

The first experiment has established that talking about object location in virtual space broadly mirrors talking about object location in real space, with the same distinction between peripersonal and extrapersonal space. In this experiment we therefore proceeded to cross, in two‐object arrays, *when* objects appeared with *where* they appeared (distance from participant).

### 3.1 Method

#### 3.1.1 Participants

Sixteen participants (eight male) were recruited from the student population. Their average age was 23.6 years (range 19–45 years), and all were right‐handed, native mono‐lingual speakers of English. Participants took part for (nominal) payment. All participants had normal or corrected‐to‐normal vision. Stereoacuity was measured using the Randot Stereotest (Stereo Optical Inc), and all participants had a threshold of at least 40 arc seconds.

#### 3.1.2 Procedure

The procedure was the same as in Experiment 1 except for two differences. Two disks always appeared. The disks either appeared at the same time or one disk appeared and then after 3 s the second disk appeared, with the first disk remaining. As in Experiment 1, participants were informed beforehand which object was the target and were told not to respond until both objects had appeared. This was a 2 (side) × 2 (distance: peripersonal or extrapersonal) × 3 (Timing: target appeared before, at the same time, or after the other disk) design. There were 72 trials. Participants were given six practice trials.

### 3.2 Results and discussion

As with Experiment 1, the percentage use of *this* was calculated for each participant, with the distance collapsed into peripersonal and extrapersonal space (Table 1). The results were analyzed in a 2 (side) × 2 (distance: peripersonal or extrapersonal) × 3 (Timing: target appeared before, at the same time, or after the other disk) anova. There was a main effect of distance, *F*(1, 15) = 19.13, *p* = .001, partial η^2^ = 0.561, with participants again using *this* more in peripersonal (*M* = 57.64%) than in extrapersonal space (*M* = 20.14%). There were no other main effects and no significant interactions. Critically, the time of appearance did not affect demonstrative choice.

These results suggest the primacy of distance over time of appearance as a determinant of demonstrative choice. Although the time of the object appearance was manipulated with a relatively long duration between appearances (3 s), relative time of appearance did not affect demonstrative choice at all. This result could be taken as an evidence that spatial distinctions are primary and temporal distinctions secondary. However, an alternative possibility is that the length of time an object is presented cancels out the possible effect of time of appearance; for that reason, we ran a third experiment where the first object disappeared prior to the presentation of the second object.

## 4 Experiment 3

### 4.1 Method

#### 4.1.1 Participants

Fifteen participants (seven male) were recruited from the student population. Their average age was 22.7 years (range 19–30 years), and all were right‐handed, native mono‐lingual speakers of English. Participants took part for (nominal) payment. All participants had normal or corrected‐to‐normal vision. Stereoacuity was measured using the Randot Stereotest (Stereo Optical Inc), and all participants had a threshold of at least 40 arc seconds.

#### 4.1.2 Procedure

The procedure for this experiment was the same as in the previous studies, with two differences. The disks either appeared together for 3 s and then disappeared, or one disk appeared for 3 s and then disappeared, and then after a gap of 3 s the second disk appeared for 3 s and then disappeared. Participants were informed beforehand what the target would be and instructed to respond only after both objects had disappeared. Instead of pointing to the objects and describing them, participants were asked to point and identify the location of where the object had been, using a demonstrative and the color of the dot (e.g., *this purple dot, that pink dot*). This was a 2 (side) × 2 (distance: peripersonal or extrapersonal) × 3 (Timing: target appeared before, at the same time, or after the other disk) design. There were 72 trials. Participants were given six practice trials.

### 4.2 Results and discussion

As with the previous experiments the percentage use of *this* was calculated for each participant, with the distances collapsed into peripersonal and extrapersonal space (Table 1). The results were analyzed in a 2 (side) × 2 (distance: peripersonal or extrapersonal) × 3 (Timing: target appeared before, at the same time, or after the other disk) anova. There was a main effect of distance, *F*(1, 14) = 29.02, *p *< .001, partial η^2^ = 0.675, with participants again using *this* more in peripersonal (*M* = 65.74%) than in extrapersonal space (*M* = 24.82%). There was no main effect of time or the side on which the target appeared. There were also no significant interactions (although there was a near significant interaction between distance and the side on which the target appeared, *F*(1, 14) = 3.88, *p* = .069, partial η^2^ = 0.217 consistent with findings in Experiment 1).

This experiment again shows that demonstratives are prioritized to make spatial distinctions, with no evidence for any influence of temporal information on demonstrative choice.

## 5 Experiment 4

The previous two experiments elicited language use to describe only one pair of objects, with the finding that demonstrative choice was unaffected by temporal manipulations. It is possible that temporal uses of demonstratives only arise when a speaker intends to contrast two objects using language (it has been argued that spatial uses of demonstratives are also contrastive; Bonfiglioli, Finocchiaro, Gesierich, Rositani, & Vescovi, 2009) though we have demonstrated that spatial differentiation of use arises in the absence of a second contrastive object (Coventry et al., 2008, 2014). In the fourth experiment we attempted to elicit contrastive temporal uses of demonstratives by presenting pairs of objects, which appeared at different times, while holding the distance constant for each pair.

### 5.1 Method

#### 5.1.1 Participants

Twenty‐one participants (three male) were recruited from the student population. Their average age was 20.0 years (range 18–24 years), and all were right‐handed, native mono‐lingual speakers of English. Participants took part for (nominal) payment. All participants had normal or corrected‐to‐normal vision. Stereoacuity was measured using the Randot Stereotest (Stereo Optical Inc), and all participants had a threshold of at least 40 arc seconds.

#### 5.1.2 Procedure

The virtual scene setup was identical to previous experiments. On each trial participants saw two disks. A third of the trials were filler trials where the two disks appeared at the same time for 3 s, before disappearing. In the remaining trials the first disk appeared for 3 s and then disappeared. After a gap of 3 s a second disk appeared for 3 s. After the second disk disappeared, a white screen appeared with an incomplete sentence. Participants were told to read out the sentence, inserting a description (color and shape, e.g., blue diamond) of each object they had seen into the two gaps, for example “I saw the *red triangle*, and the *green star*.” They were told that the sentences would vary but that the format remained the same so that each participant had a similar experience. There were three different sentences (referred to as “this,” “that,” and “the” trials first mention respectively, hereafter):

I saw this _________, and that _________

I saw that _________, and this _________

I saw the _________, and the _________

Participants were given no instructions as to which object description (first seen or second seen) to insert in each gap.

This was a 2 (side: left or right) × 2 (distance: peripersonal or extrapersonal) × 3 (sentence type: *this, that, the*) design. The trials where both objects appeared together were filler trials. There were 108 trials. Participants were given six practice trials.

### 5.2 Results and discussion

The filler trials, where two objects appeared at the same time, were omitted from the analysis. On each trial, participants saw two objects, one appearing after the other. They completed a sentence (with two gaps) with the objects. Once the participant had chosen an object to fill the first sentence gap, the choice for the second gap is fixed. For the main analysis we, therefore, analyzed only the choice for the first sentence gap. For each participant we calculated the percentage of times the *first object that appeared* was chosen to fill the *first gap* in the sentence, for each condition. The results were analyzed in a 2 (side: left or right) × 2 (distance: peripersonal or extrapersonal) × 3 (sentence type: *this* first slot*, that* first slot*, the*) anova.

The most striking result is that participants overwhelmingly chose to use the first object that had appeared, in the first sentence gap (84.6% of the time) (Table 2). A one‐sample *t* test comparing the percentage of times the first object was chosen to fill the first gap (compared to the 50% that would be expected by chance) revealed that this was significant, *t*(20) = 9.04, *p *< .001, *d* = 1.97. This was significant for each condition (*p *< .01, with the smallest effect size of the conditions being *d* = 0.72), showing that participants were sensitive to the order of presentation when recalling objects.

**Table 2:** Mean percentage of times the first object that appeared was chosen to fill the first gap in the sentence in Experiment 4, in each condition across peripersonal and extrapersonal space, split by side

Sentence Type	Target Position	Peripersonal (25–75 cm)		Extrapersonal (100–150 cm)	
		*M*	*SEM*	*M*	*SEM*
This	Left	92.38	2.91	92.06	3.38
	Right	81.59	6.39	79.68	6.49
That	Left	88.16	4.59	90.63	3.33
	Right	76.19	7.50	73.97	7.30
The	Left	91.90	3.59	92.86	2.95
	Right	79.05	6.43	76.98	6.55

**Figure 4:**
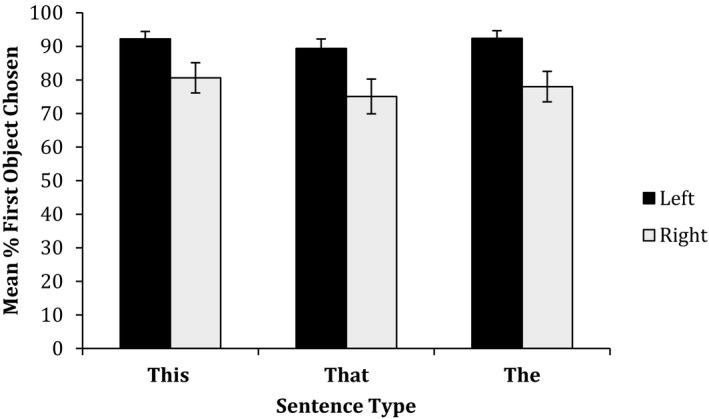
Mean percentage of times the *first object that appeared* was chosen to fill the *first gap* in the sentence in Experiment 4. Error bars represent standard errors of the mean.

Despite the sensitivity to presentation order, the main anova analysis revealed there was no effect of sentence type (demonstrative use) on participants’ choice, *F*(2, 40) = 1.50, *p* = .236, partial η^2^ = 0.070. The six distances were collapsed into peripersonal and extrapersonal space. There was no main effect of distance, *F*(1, 40) = 0.17, *p* = .681, partial η^2^ = 0.009. There was a main effect of side, *F*(1, 40) = 5.33, *p* = .032, partial η^2^ = 0.210, with participants choosing to use the *first object* in the *first gap* more often when that first object appeared on the left‐hand side (see Fig. 4). None of the interactions were significant (though again there was a near significant interaction between side (left/right) and distance (peripersonal/extrapersonal), *F*(1, 40) = 3.13, *p* = .092, partial η^2^ = 0.135, consistent with the pattern of data in previous experiments).

When asked to recall a pair of objects, it is clear that participants are affected by the order in which those objects were presented, choosing overwhelmingly to name first the objects that were presented first. However, participants were not affected by the demonstrative in the sentence completion. Participants are clearly sensitive to the temporal order in which the objects appeared but temporal order did not affect demonstrative use. These results seem to indicate a much weaker relationship between demonstratives and temporal situations.

The preference for choosing to name the *first object* first more often when that first object appeared on the left‐hand than the right‐hand side seems likely linked to the English reading system, of reading left to right.

## 6 Further combined analysis

**Figure 5:**
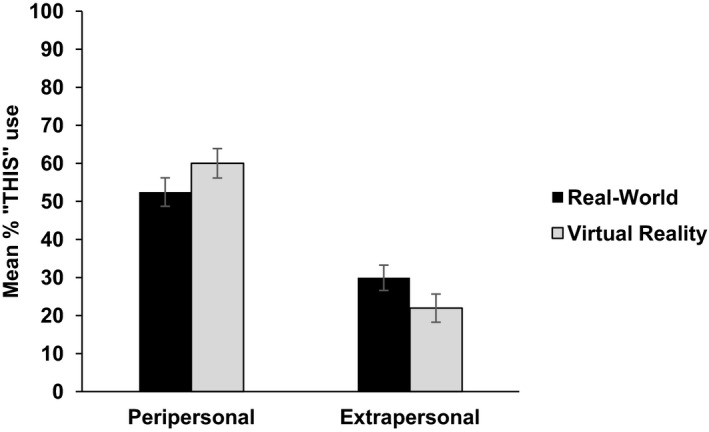
Significant interaction between distance and environment. Error bars represent standard errors of the mean.

The results from Experiment 1 revealed a trend toward an interaction when comparing the results of percentage *this* use in the real‐ versus virtual world studies, but the sample size in Experiment 1 increases the risk of a Type II error. For that reason we carried out a combined analysis by comparing the combined results from the single‐object condition results from Experiment 1, and the target appearing first condition results from Experiments 2 and 3 (which are closest to the conditions in the real‐world experiments) to the results from Coventry et al.'s (2014) language experiments (Experiments 1, 3, and 5). A 2 (distance: peripersonal or extrapersonal) × 2 (Environment: real vs. VR) anova again found no main effect of environment, *F*(1, 106) = 0.002, *p* = .961 partial η^2^ **< 0.001 and the expected significant effect of distance, *F*(1, 106) = 87.577, *p *< .001 partial η^2^ = 0.452. However, this analysis revealed a small but significant interaction between distance and environment, *F*(1, 106) = 5.762, *p* = .018 partial η^2^ = 0.052, illustrated in Fig. 5.

There was a higher usage of *this* in VR peripersonal space compared to the real‐world studies and a lower usage in extrapersonal space. Coventry et al. (2014) show that there is a direct relationship between object location memory and individual choice of demonstrative. The memory for object location directly relates to the perceived location. As discussed, there is a considerable amount of evidence that suggests that distance perception in VR is underestimated (see Renner et al., 2013, for a review). If this is the case we would, therefore, expect to see an increased use of *this* in VR compared to the real world, as objects are perceived closer than they really are. This would seem to explain the results for objects shown in peripersonal space, where we see a higher use of *this* in VR; however, it does not explain the drop in usage found in extrapersonal space, suggesting that the story in the key areas of peripersonal space and the area just beyond our reach is more complicated.

Previous research (see Creem‐Regehr et al., 2015; Renner et al., 2013) has identified a number of factors such as perceptual‐motor feedback, the use of self‐avatars, and familiarization prior to the study that can be used to improve distance estimation. We applied several of these aspects to our study in order to maximize the accuracy of participants’ distance perception in order to give the best chance to produce results in VR that matched those in our real‐world studies. While we did find a very consistent and similar pattern of results to those in the real world, there were nevertheless some differences that suggest participants showed some underestimation of distances in peripersonal space and overestimation in extrapersonal space that fed into and affected demonstrative choice.

## 7 General discussion

The present experiments were designed to pit where objects appear in a spatial array against when they appear in order to establish if space, time, or a combination of the two affects how one uses demonstratives to refer to objects. In order to do so, we adopted a VR method to control object presentation. To our knowledge this is the first time the mapping between spatial language and spatial perception has been investigated in a VR setting. We, therefore, consider the method prior to consideration of the theoretical implications of our results.

The first experiment tested whether VR as a method produces language data similar to data produced when one talks about real objects in real space. Our results reveal that broadly speaking VR produces language data consistent with that produced in real space (Coventry et al., 2008, 2014 and consistent with the perception of space; Berti & Rizzolatti, 2002). There is a clear distinction between peripersonal and extrapersonal space, with a higher use of *this* in peripersonal space. Our combined analysis, however, reveals a small but significant interaction; there is a higher use of *this* in peripersonal space in VR compared to the real world, but a lower use in extrapersonal space, suggesting under and overestimates of distance, respectively.

We took account of the previous research of VEs and included a number of aspects in our study designed to reduce as much as possible the well‐known underestimation effect of distance judgments. In the case of the peripersonal space results, this was clearly not sufficient to reduce the underestimation, and our results here are very consistent with previous research (Renner et al., 2013), though it should be noted that the interaction effect was small. The overall results, and in particular those from peripersonal space, however, lend support for the strong link between spatial perception and our use of spatial language, demonstratives, that extend into VEs. As the first study examining the mapping between spatial language and spatial perception in VR, it offers a promising methodology in which to investigate language, and further studies may shed light on the issue of distance estimates in VEs.

While there is some indication that exact metric estimations may differ from the real world (as found in many studies, Renner et al., 2013), it is clear that VR provides a useful tool for the study of language, offering the possibility to manipulate the environment in ways not possible in the real‐world.

Our primary goal was to establish if space, time, or a combination of the two affects how one uses demonstratives to refer to objects. Experiments 2 and 3 crossed distance with time. The results show that distance affects demonstrative choice, but time of appearance does not. In Experiment 4 we attempted to illicit contrastive temporal use by presenting two temporally separated objects and a sentence completion task, keeping distance constant for each pair. While participants were sensitive to the presentation order of the objects, their completion of sentences was unaffected by the demonstratives. Just as spatial demonstratives appear in the child's lexicon earlier than other uses of the same terms (Clark, 2003; Küntay & Özyürek, 2006), our results show that spatial uses of demonstratives are prioritized in processing and language choice (what is learned first is processed first; cf. Juhasz, 2005). The temporal manipulations in the experiments did not impact upon demonstrative choice at all.

The dominance of space over time across experiments in demonstrative use may be linked to the fact that objects in all the experiments were physically presented in space at varying distances from the body. It is possible that the physical presence of objects entails that physical distance is the primary driver for processing of objects. It is well established that there are two different brain systems that represent near/peripersoanl space on the one hand (reachable space in front of the body) and far/extrapersonal space on the other (space outside of immediate grasp/contact) (Berti & Rizzolatti, 2002; Làdavas, 2002; Legrand, Brozzoli, Rossetti, & Farnè, 2007). Evidence from fMRI studies identifies the posterior parietal cortex, specifically the superior parieto‐occipital cortex (SPOC) and the intraparietal sulcus, as the possible neural substrate for peripersonal space (e.g., Gallivan, McLean, & Culham, 2011; Makin, Holmes, & Zohary, 2007). Moreover, it has been shown that SPOC encodes reachable space even during passive viewing of objects, consistent with the view that motor affordances are automatically processed in this reach‐selective region (Gallivan, Cavina‐Pratesi, & Culham, 2009). This automatic brain coding of distance makes distance information readily available to use for communication, and it may well result in the primacy of space over time in these circumstances.

While space may dominate, it is also important to note that spatial and temporal factors do not appear to interact in the use of demonstratives. In other words, when demonstratives are used spatially, they do not appear to be influenced by temporality. This raises the possibility that temporal and spatial uses of demonstratives might be dissociated. Such a possibility is not without precedent. Kemmerer (2005) has shown that the English use of prepositions—other terms that can be used spatially (e.g., *on the table*) and temporally (e.g., *on Monday*)—can be selectively impaired. He reports a double dissociation between performance on tests assessing spatial and temporal prepositions with four patients with perisylvian lesions, supporting the view that spatial and temporal terms in language may be represented independently.

While the time of appearance does not constrain demonstrative choices when distance is also manipulated, there were some effects linked to action and attention that merit closer inspection in future studies. In Experiments 1, 3, and 4, there was a tendency for *this* to be preferred (over *that*) when describing an object located on the right side in peripersonal space more than for an object on the left side—but this pattern was reversed in extrapersonal space. This confirms that demonstrative choice is affected by the ease of the mapping between the object location and deictic pointing when the object is reachable. As Diessel (2006) has noted, in some languages it is obligatory to point when using demonstratives to refer to objects in the physical world (*Goemai*, Hellwig, 2003; *Kilivili*, Senft, 2004), and deictic gestures also seem to be a predictor of language acquisition (Iverson & Goldin‐Meadow, 2005). In contrast, when the hand cannot directly reach an object, working attentionally from left to right seems to take over, and this is likely to be tied to writing systems (Bergen & Lau, 2013; Shaki, Fischer, & Petrusic, 2009); testing speakers of other languages on the memory game paradigm would allow this account to be properly empirically evaluated. The order, in English, of reading left to right may also account for the effects found in Experiment 4, where participants chose to mention the *first object* first more often when that first object appeared on the left‐hand than the right‐hand side.

Overall, the results show that there is primacy of spatial language over non‐spatial uses of those same terms. However, two points should be noted. First, the failure to find evidence for temporal uses of demonstratives in our experiments means it is premature to argue that the data provide support for conceptual metaphor theory (e.g., Casasanto & Boroditsky, 2008; Lakoff & Johnson, 1980). Such a conclusion would require showing that spatial uses are prioritized over temporal uses and further that temporal uses are structured by space. Second, as discussed earlier, the overtly spatial nature of the task may have reduced the likelihood of participants using demonstratives temporally. Future studies would do well to employ methods that magnify the temporal dimensions of a task while diminishing spatial dimensions to increase the chances of demonstratives being used temporally. For example, this could be done using the haptic modality rather than the visual modality (see Cai & Connell, 2015) and/or presenting single sounds (rather than objects) localized in space with longer time intervals between sound occurrences. Nevertheless, using language choice as a measure when space and time are experimentally crossed, we think, provides a useful complementary method to address their relationship.

## Note

1 It should be noted that *this* is not always used in peripersonal space and *that* is also not always used in extrapersonal space; there are other parameters that also affect demonstrative usage in addition to perceptual space; see Coventry et al. (2014) for discussion.
